# Endothelin-1 as a Biomarker of Idiopathic Pulmonary Fibrosis and Interstitial Lung Disease Associated with Autoimmune Diseases

**DOI:** 10.3390/ijms24021275

**Published:** 2023-01-09

**Authors:** Verónica Pulito-Cueto, Fernanda Genre, Raquel López-Mejías, Víctor Manuel Mora-Cuesta, David Iturbe-Fernández, Virginia Portilla, María Sebastián Mora-Gil, Javier Gonzalo Ocejo-Vinyals, Oreste Gualillo, Ricardo Blanco, Alfonso Corrales, Iván Ferraz-Amaro, Santos Castañeda, José Manuel Cifrián Martínez, Belén Atienza-Mateo, Sara Remuzgo-Martínez, Miguel Ángel González-Gay

**Affiliations:** 1 Research Group on Genetic Epidemiology and Atherosclerosis in Systemic Diseases and in Metabolic Bone Diseases of the Musculoskeletal System, Instituto de Investigación Marqués de Valdecilla, Avenida Cardenal Herrera Oria s/n, Lab. 201/202, 39011 Santander, Spain; 2 Pneumology Department, Hospital Universitario Marqués de Valdecilla, 39008 Santander, Spain; 3 Rheumatology Department, Hospital Universitario Marqués de Valdecilla, 39008 Santander, Spain; 4 Department of Immunology, Hospital Universitario Marqués de Valdecilla, 39008 Santander, Spain; 5 SERGAS (Servizo Galego de Saude) and IDIS (Instituto de Investigación Sanitaria de Santiago), NEIRID Lab. (Neuroendocrine Interactions in Rheumatology and Inflammatory Diseases), Research Laboratory 9, Santiago University Clinical Hospital, 15706 Santiago de Compostela, Spain; 6 Department of Rheumatology, Hospital Universitario de Canarias, 38320 Santa Cruz de Tenerife, Spain; 7 Rheumatology Department, Hospital Universitario de la Princesa, IIS-Princesa, Cátedra UAM-ROCHE EPID Futuro, Universidad Autónoma de Madrid, 28006 Madrid, Spain; 8 School of Medicine, Universidad de Cantabria, 39011 Santander, Spain; 9 Department of Medicine and Psychiatry, Universidad de Cantabria, 39011 Santander, Spain; 10 Cardiovascular Pathophysiology and Genomics Research Unit, School of Physiology, Faculty of Health Sciences, University of the Witwatersrand, Johannesburg 2000, South Africa

**Keywords:** interstitial lung disease, idiopathic pulmonary fibrosis, interstitial lung disease associated with autoimmune diseases, biomarker, endothelin-1

## Abstract

The aim of this study was to determine the role of endothelin-1 (ET-1), a molecule involved in multiple vascular and fibrosing abnormalities, as a biomarker of interstitial lung disease (ILD), as well as its use for the differential diagnosis between idiopathic pulmonary fibrosis (IPF) and ILD associated with autoimmune diseases (AD-ILD), using a large and well-defined cohort of patients with ILD. A total of 112 patients with IPF, 91 patients with AD-ILD (28 rheumatoid arthritis (RA), 26 systemic sclerosis, 20 idiopathic inflammatory myositis and 17 interstitial pneumonia with autoimmune features) and 44 healthy controls were included. ET-1 serum levels were determined by enzyme-linked immunosorbent assay. A significant increase in ET-1 levels was found in patients with IPF compared to controls. Likewise, AD-ILD patients also showed higher ET-1 levels than controls when the whole cohort was stratified by the type of AD. Similar ET-1 levels were found in IPF and AD-ILD patients, regardless of the underlying AD. Interestingly, increased ET-1 levels were correlated with worse lung function in IPF and RA-ILD patients. Our study supports that serum ET-1 may be useful as a biomarker of ILD, although it could not help in the differential diagnosis between IPF and AD-ILD. Moreover, ET-1 levels may be associated with ILD severity.

## 1. Introduction

Idiopathic pulmonary fibrosis (IPF) and interstitial lung disease associated with autoimmune diseases (AD-ILD) are two relevant groups of chronic parenchymal pulmonary disorders encompassed under the term interstitial lung disease (ILD) [1]. IPF is the most frequent and severe ILD triggered by an unknown cause, whereas AD-ILD are constituted by different systemic rheumatic diseases characterized by a dysregulation of the immune system in which lung involvement is a common extraarticular manifestation [1,2,3,4]. In this regard, it was previously reported that among the spectrum of AD, ILD is more frequently found in patients with rheumatoid arthritis (RA), systemic sclerosis (SSc) and idiopathic inflammatory myositis (IIM) [3]. Interestingly, there is also a significant prevalence of patients with interstitial pneumonia with autoimmune features (IPAF), characterized by the presence of ILD and clinical, serological, radiographic and/or histological characteristics that suggest the presence of an underlying AD, but who do not fulfill the diagnostic criteria for any specific AD [3,5]. An increased risk of morbidity and mortality is found in ILD patients, with IPF patients having a worse prognosis [1,2,3,4,6,7]. Consequently, an early and precise diagnosis of ILD is crucial for the better management and adequate treatment of these patients. However, its diagnosis is often difficult and delayed, constituting a significant concern for clinicians [1,2].

Endothelin-1 (ET-1) is a molecule mainly produced by the vascular endothelium that plays a key role in vascular tone homeostasis [8,9]. In this regard, elevated ET-1 levels have been associated with vasoconstriction, vascular hypertrophy, inflammation and pulmonary fibrosis [10,11,12,13,14]. Accordingly, it is involved in the pathogenesis of numerous acute and chronic lung diseases, including asthma, chronic obstructive pulmonary disease, lung malignancies, acute respiratory distress syndrome, pulmonary arterial hypertension and parenchymal lung diseases [10,13,14]. In particular, a profibrotic role of ET-1 has been described in patients with IPF [15,16]. In addition, it has been suggested as a potential biomarker of SSc-ILD [17,18,19,20,21]. However, further studies in a large cohort of IPF patients and in different types of AD-ILD are required to shed light on this issue. 

In view of the above, in this study we aimed to determine the role of serum ET-1 as a biomarker of ILD, as well as its use for the differential diagnosis between IPF and AD-ILD, using a large and well-defined cohort of patients with ILD.

## 2. Results

### 2.1. ET-1 as a Biomarker of ILD

**Figure 1:**
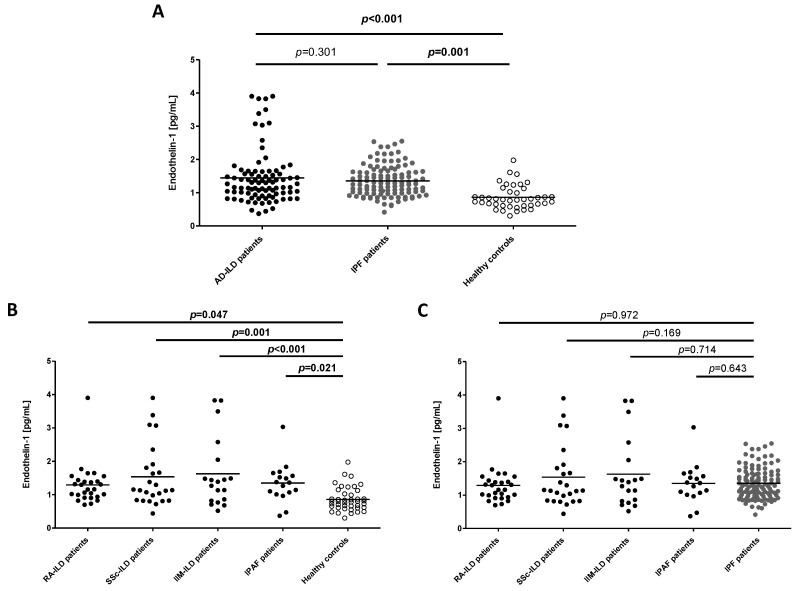
Differences in ET-1 serum levels in all the individuals included in the study. (**A**) Comparison of ET-1 levels between all the study groups; (**B**) Comparison of ET-1 levels between each type of AD-ILD patient and healthy controls; (**C**) Comparison of ET-1 levels between each type of AD-ILD and IPF patient. Horizontal bars indicate the mean value of each study group. Significant results are highlighted in bold.

First, we studied the role of ET-1 in the pathogenesis of ILD. In this regard, a statistically significant increase in ET-1 serum levels in patients with ILD (both AD-ILD and IPF) compared to healthy controls was disclosed (Figure 1A). In particular, the mean ET-1 level in patients with AD-ILD and IPF was 1.44 ± 0.81 pg/mL and 1.35 ± 0.46 pg/mL, respectively, whereas in controls it was 0.86 ± 0.35 pg/mL. This significant increase in ET-1 serum levels was also found when the whole cohort of AD-ILD patients was stratified according to the different types of AD (Figure 1B, Table S1).

**Figure 2:**
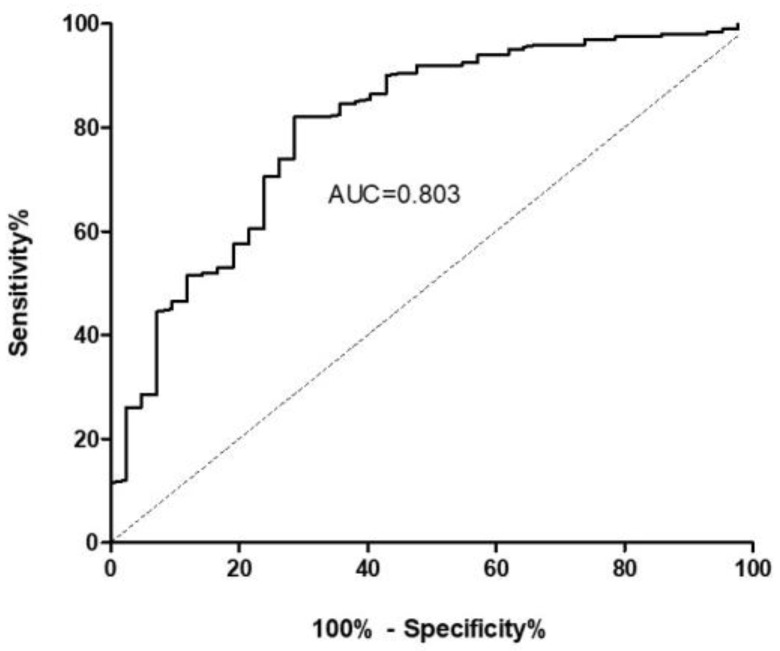
Receiver operating characteristic (ROC) curve indicating the power of serum ET-1 for the diagnosis of patients with ILD. The solid line represents the ROC curve that joins the different cut-off points. The dotted line drawn from point 0 to 100 shows the diagonal reference line. AUC = Area under the curve.

To confirm the usefulness of ET-1 to discriminate patients with ILD from healthy controls, receiver operating characteristic (ROC) curve was performed (Figure 2). The area under the curve was 0.803 (95% confidence interval: 0.728–0.878). Based on this, the optimal cut-off value for ET-1 levels showing the best sensitivity (82.0%) and specificity (71.4%) was 0.88 pg/mL.

### 2.2. ET-1 for the Differential Diagnosis between ILD Patients

Secondly, we analyzed whether ET-1 may be useful for the differential diagnosis between AD-ILD and IPF patients. In this regard, similar ET-1 levels were found in these patients (Figure 1A). In fact, no significant differences were observed between the ET-1 levels of patients with IPF and each subtype of AD (RA-ILD, SSc-ILD, IIM-ILD or IPAF) (Figure 1C, Table S1). 

### 2.3. Association of ET-1 with Clinical Characteristics of Patients with ILD

**Figure 3:**
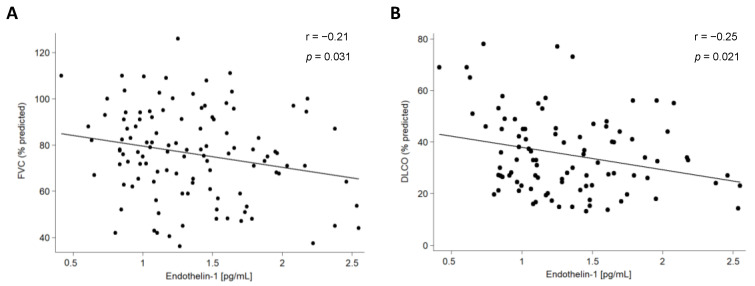
Partial correlation between ET-1 serum levels and FVC (**A**) and DLCO (**B**) in IPF patients.

**Figure 4:**
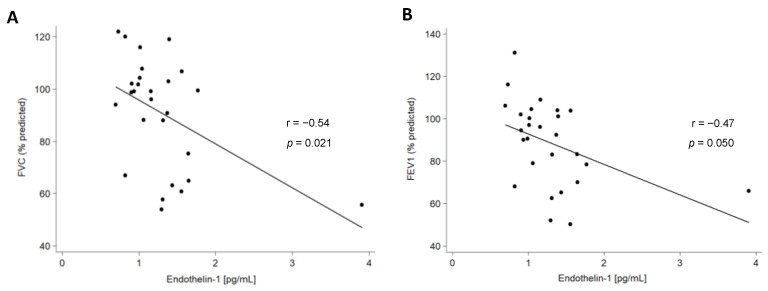
Partial correlation between ET-1 serum levels and FVC (**A**) and FEV1 (**B**) in RA-ILD patients.

We also assessed the relationship of ET-1 serum levels with the clinical characteristics of all the patients included in this study. Regarding pulmonary function tests (PFTs), a negative correlation between ET-1 serum levels and both forced vital capacity (FVC) and diffusing capacity of the lungs for carbon monoxide was disclosed in IPF patients (Figure 3, Table 1). Likewise, a negative correlation between ET-1 serum levels and FVC and forced expiratory volume in one second (FEV1) was found in AD-ILD patients (Table 1). This finding was specifically related to RA-ILD patients (Figure 4, Table S2).

No significant differences were found between the ET-1 levels and other clinical characteristics of patients with IPF, the whole cohort of AD-ILD patients or each type of AD-ILD patient (Table 1 and Table S2).

## 3. Discussion

The study of biomarkers as additional tools in the clinical practice for the diagnosis and severity of ILD has aroused great interest in the last years [17,22,23,24,25,26,27,28,29]. In this regard, the role of ET-1 in the pathogenesis of a large and well-defined cohort of ILD patients remains to be investigated in depth. Given that this molecule is involved in characteristic processes of ILD, such as vascular dysfunction, inflammation and pulmonary fibrosis [2,11,12,13,14,30], ET-1 can constitute a promising candidate biomarker of this complex group of diseases. 

Our study supports that ET-1 plays a relevant role in the pathogenesis of ILD, owing to the fact that ET-1 serum levels were increased in both IPF and AD-ILD patients compared to healthy controls. This same finding was previously observed in a small cohort of IPF patients [15], whereas there are no previous studies regarding the role of ET-1 in AD-ILD patients as a whole. Interestingly, we also found an increase in ET-1 serum levels when each AD-ILD was analysed separately. In this respect, previous studies obtained similar results for SSc-ILD patients [19,21]. Thereby, our results broaden the relevance of ET-1 not only in SSc-ILD but also, for the first time, in RA-ILD, IIM-ILD and IPAF. It is plausible to think that the underlying inflammation and/or fibrosis in our patients are responsible for the high ET-1 levels, considering that inflammatory mediators and hypoxia have been previously associated with ET-1 overproduction [8,16,30,31]. Taking into account the role of ET-1 in ILD disclosed in our study and that previously reported in other lung diseases [10], ET-1 may constitute a biomarker of different lung diseases, not exclusively those associated with pulmonary fibrosis. Therefore, the measurement of ET-1 serum levels together with standardized complementary tests and clinical examination may contribute to the early diagnosis of ILD. In particular, concentrations of ET-1 higher than 0.88 pg/mL may differentiate ILD from healthy subjects. Furthermore, these results point out that ET-1 may constitute a target for therapeutic strategies in ILD. In this regard, bosentan, a potent dual antagonist of ET-1 receptors, is currently indicated for the treatment of pulmonary arterial hypertension and for the prevention of recurrent digital ulcers in patients with SSc [32]. Accordingly, our data support the potential use of bosentan in patients with ILD, specifically in IPF and AD-ILD. 

We also disclosed, for the first time, a significant association of higher levels of ET-1 with lower FVC and DLCO in IPF patients, probably due to the greater severity of ILD in these patients. In the same line, ET-1 was negatively associated with FVC and FEV1 in the whole cohort of AD-ILD patients. This was confirmed in RA-ILD patients in particular. In keeping with this data, an inverse correlation between ET-1 and altered PFTs has been reported in patients with chronic obstructive pulmonary disease [33]. The relationship of ET-1 with the severity of ILD in RA-ILD and IPF would be consistent with the poor prognosis and other similarities described between patients with these conditions, with it not being uncommon that individuals initially defined as having IPF may be eventually diagnosed with RA-ILD [3,26].

Since the accurate diagnosis of ILD implies different therapeutic strategies influencing the management of these patients [1,2,3,34], the identification of biomarkers for the differential diagnosis between IPF and other ILD is relevant. In this regard, previous studies from our group and others have revealed the potential relevance of biomarkers such as endothelial progenitor cells and advanced glycosylated end-products to discriminate between IPF and ILD associated with connective tissue diseases [26,35]. In a similar manner, the glycoprotein gremlin-1 has been proposed for the differential diagnosis of IPF compared to non-IPF ILD [36]. Interestingly, it has been disclosed that telomere shortening and increased DNA damage could constitute potential biomarkers in this context [37,38]. In particular, telomere length may help to differentiate between patients with IPF and those with idiopathic non-specific interstitial pneumonia and sarcoidosis [39]. Despite these findings shedding some light on the differential diagnosis of IPF compared to other types of ILD, more studies addressing this concern are needed. In this regard, our study suggests that ET-1 could not help to distinguish between IPF and AD-ILD patients.

We acknowledge that the present study has some limitations. Given the cross-sectional design of our study, we cannot evaluate whether the correlation with disease score changes as the disease progresses and the autoimmune patients develop lung disease. Additionally, replication of our results in a different cohort may help validate our findings.

## 4. Materials and Methods

### 4.1. Patients and Healthy Controls 

A total of 203 patients fulfilling the American Thoracic Society/European Respiratory Society classification and diagnosis criteria for ILD [40,41] were recruited for this study. The whole cohort of ILD patients was composed of 112 patients with IPF [6] and 91 patients with AD-ILD (28 RA, 26 SSc, 20 IIM, according to the applicable diagnostic or classification criteria established by the American College of Rheumatology/European League Against Rheumatism for each AD [42,43,44,45,46] and 17 IPAF [5]). Demographic and clinical features including sex, age, smoking history, packs of cigarettes per year, PFTs and high-resolution computed tomography (HRCT) patterns stratified according to the criteria for usual interstitial pneumonia (UIP) pattern of the Fleischner Society: UIP pattern, probable UIP pattern, indeterminate for UIP pattern and features most consistent with an alternative diagnosis [47] were collected from the patients (Table 2 and Table S3).

In addition, 44 healthy controls, without history of any chronic inflammatory or lung disease, were also included in this study. Their mean age ± standard deviation (SD) was 51.0 ± 11.7 years, 40.9% of them were men, and 33.3% were smokers.

All the individuals were recruited from the Pneumology and Rheumatology departments of Hospital Universitario Marqués de Valdecilla, Santander, Spain and each individual gave their written informed consent to be included in the study. The procedures followed were in accordance with the ethical standards of the approved guidelines and regulations according to the World Medical Association Declaration of Helsinki. The research protocol was approved by the Ethics Committee of clinical research of Cantabria, Spain (2016.092).

### 4.2. ET-1 Serum Levels Determination

ET-1 levels were measured in serum samples from all the individuals by a commercial enzyme-linked immunosorbent assay (Endothelin-1 Quantikine Kit, DET100, Bio-Techne R&D Systems S.L.U., Minneapolis, MN, USA) in accordance with the manufacturer’s instructions. Samples were analyzed in duplicate. ET-1 levels were quantified using a four-parameter logistic curve fit suitable for calculating concentrations from symmetrical sigmoidal calibrators through MyAssays Ltd 2021 online software. 

### 4.3. Statistical Analysis

Data were reported as the number of individuals (n) and percentage (%) for categorical variables and mean ± SD for continuous variables. Differences in ET-1 serum levels were compared between the study groups by ANOVA, adjusting *p* values for sex, age and smoking history. Number of packs of cigarettes per year was also included as a potential confounding factor when differences between IPF and AD-ILD patients were evaluated. When significant differences between groups were obtained, ROC analysis and the optimal cut-off value (the higher value obtained from the formula sensitivity% + specificity% − 1) of ET-1 for the diagnosis of ILD was performed. In addition, the association between ET-1 levels and clinical characteristics of all the patients were calculated by ANOVA or Pearson’s partial correlation coefficient (r), when appropriate, adjusting *p* values for the potential confounding factors previously mentioned. *p*-values ≤ 0.05 were considered statistically significant. Statistical analysis was carried out with STATA statistical software 12/SE (Stata Corp., College Station, TX, USA).

## 5. Conclusions

Our study supports that serum ET-1 may be useful as a biomarker of ILD, although it could not help in the differential diagnosis between IPF and AD-ILD. Moreover, ET-1 levels may be associated with ILD severity. Translated into clinical practice, the determination of high serum ET-1 levels may indicate the presence of ILD itself.

## Figures and Tables

**Table 1:** Association between ET-1 serum levels and clinical characteristics of the patients included in the study.

		IPF Patients		AD-ILD Patients	
Variable		*r*	*p*	*r*	*p*
FVC (% predicted)		**−0.21**	**0.031**	**−0.35**	**0.002**
FEV1 (% predicted)		−0.15	0.119	**−0.30**	**0.010**
DLCO (% predicted)		**−0.25**	**0.021**	−0.16	0.257
Variable	Category	Mean ± SD [pg/mL]	*p*	Mean ± SD [pg/mL]	*p*
HRCT pattern	UIP	1.35 ± 0.46	-	1.49 ± 0.83	0.354
	NSIP	-		1.30 ± 0.65	

AD: autoimmune diseases; DLCO: diffusing capacity of the lungs for carbon monoxide; FEV1: forced expiratory volume in one second; FVC: forced vital capacity; HRCT: high-resolution computed tomography; ILD: interstitial lung disease; IPF: idiopathic pulmonary fibrosis; NSIP: non-specific interstitial pneumonia; SD: standard deviation; UIP: usual interstitial pneumonia. UIP category includes patients with both UIP and probable UIP pattern for AD-ILD patients. Significant results are highlighted in bold.

**Table 2:** Demographic and clinical characteristics of the patients included in the study.

Characteristic	IPF Patients (n = 112)	AD-ILD Patients (n = 91)
Sex (men/women), n (%)	93/19 (83.0/17.0)	48/43 (52.7/47.3)
Age at the time of the study (years), mean ± SD	64.5 ± 6.3	61.1 ± 9.2
Smoking history, n (%)	94 (83.9)	60 (65.9)
Packs of cigarettes per year, mean ± SD	32.6 ± 21.0	28.1 ± 24.2
Pulmonary function tests		
FVC (% predicted), mean ± SD	76.2 ± 19.3	80.4 ± 25.7
FEV1 (% predicted), mean ± SD	77.9 ± 19.5	79.1 ± 24.9
DLCO (% predicted), mean ± SD	35.2 ± 15.3	39.6 ± 17.6
HRCT pattern		
UIP, n (%)	112 (100.0)	33 (39.8)
Probable UIP, n (%)	-	10 (12.0)
Indeterminate for UIP pattern, n (%)	-	2 (2.4)
Features most consistent with an alternative diagnosis		
NSIP, n (%)	-	34 (41.0)
Non-NSIP, n (%)	-	4 (4.8)

AD: autoimmune diseases; DLCO: diffusing capacity of the lungs for carbon monoxide; FEV1: forced expiratory volume in one second; FVC: forced vital capacity; HRCT: high-resolution computed tomography; ILD: interstitial lung disease; IPF: idiopathic pulmonary fibrosis; NSIP: non-specific interstitial pneumonia; SD: standard deviation; UIP: usual interstitial pneumonia. AD-ILD group includes patients with rheumatoid arthritis (n = 28), systemic sclerosis (n = 26), idiopathic inflammatory myositis (n = 20) and interstitial pneumonia with autoimmune features (n = 17).

## Data Availability

All data generated or analyzed during this study are included in this published article and in the Supplementary Materials.

## Supplementary Materials

The following supporting information can be downloaded at: https://www.mdpi.com/article/10.3390/ijms24021275/s1.
