# Supplementary material for: Endothelin-1 as a Biomarker of Idiopathic Pulmonary Fibrosis and Interstitial Lung Disease Associated with Autoimmune Diseases
Source: Int J Mol Sci. 2023 Jan 9;24(2):1275. doi: 10.3390/ijms24021275 (PMC9862125; doi:10.3390/ijms24021275)
Supplement: Supplementary file 1 [file ijms-24-01275-s001.zip › ijms-2083135-supplementary.pdf]

Table S1. ET-1 serum levels in all the individuals included in the study.

	**ET-1 [pg/mL] (mean ± SD)**
IPF patients	1.35 ± 0.46
AD-ILD patients	1.44 ± 0.81
RA-ILD	1.29 ± 0.60
SSc-ILD	1.54 ± 0.93
IIM-ILD	1.63 ± 1.05
IPAF	1.35 ± 0.59
Healthy controls	0.86 ± 0.35

AD: autoimmune diseases; IIM: idiopathic inflammatory myositis; ILD: interstitial lung disease; IPAF: interstitial pneumonia with autoimmune; RA: rheumatoid arthritis; SD: standard deviation; SSc: systemic sclerosis.

Table S2. Association between ET-1 serum levels and clinical characteristics of each type of AD-ILD patients.

		RA-ILD		SSc-ILD		IIM-ILD		IPAF	
Variable		*r*	*p*	*r*	*p*	*r*	*p*	*r*	*p*
FVC (% predicted)		**-0.54**	**0.021**	-0.43	0.082	0.01	0.997	-0.03	0.928
FEV1 (% predicted)		**-0.47**	**0.050**	-0.37	0.144	-0.01	0.979	0.05	0.872
DLCO (% predicted)		-0.37	0.260	-0.49	0.148	0.47	0.239	-0.60	0.068
Variable	Category	*Mean ± SD*	*p*	*Mean ± SD*	*p*	*Mean ± SD*	*p*	*Mean ± SD*	*p*
HRCT pattern	UIP	1.37 ± 0.73	0.865	1.64 ± 0.75	0.478	1.78 ± 1.25	0.140	1.36 ± 0.76	0.579
	NSIP	1.21 ± 0.32		1.35 ± 0.89		1.28 ± 0.55		1.39 ± 0.25	

AD: autoimmune diseases; DLCO: diffusing capacity of the lungs for carbon monoxide; FEV1: forced expiratory volume in one second; FVC: forced vital capacity; HRCT: high-resolution computed tomography; IIM: idiopathic inflammatory myositis; ILD: interstitial lung disease; IPAF: interstitial pneumonia with autoimmune features; NSIP: non-specific interstitial pneumonia; RA: rheumatoid arthritis; SD: standard deviation; SSc: systemic sclerosis; UIP: usual interstitial pneumonia. UIP category includes patients with both UIP and probable UIP pattern. Significant results are highlighted in bold.

Table S3. Demographic and clinical characteristics of AD-ILD patients according to the underlying AD.

	**RA-ILD**	**SSc-ILD**	**IIM-ILD**	**IPAF**
Sex (men/women), n (%)	16/12	11/15	8/12	13/4
Age at the time of the study (years), mean ± SD	65.7 ± 9.3	60.0 ± 7.3	56.8 ± 9.2	60.2 ± 8.9
Smoking history, n (%)	19 (73.1)	14 (63.6)	14 (70.0)	13 (76.5)
Packs of cigarettes per year, mean ± SD	36.1 ± 22.3	19.4 ± 12.2	17.2 ± 12.6	39.5 ± 36.4
Pulmonary function tests				
FVC (% predicted), mean ± SD	88.4 ± 24.4	79.7 ± 25.3	69.9 ± 24.0	80.0 ± 28.0
FEV1 (% predicted), mean ± SD	86.5 ± 22.7	77.9 ± 24.4	69.1 ± 24.8	79.8 ± 27.1
DLCO (% predicted), mean ± SD	39.5 ± 17.4	36.3 ± 17.2	39.5 ± 15.5	42.1 ± 19.7
HRCT pattern				
UIP, n (%)	15 (57.7)	6 (25.0)	5 (26.3)	7 (50.0)
Probable UIP, n (%)	2 (7.7)	1 (4.2)	4 (21.1)	3 (21.4)
Indeterminate for UIP pattern, n (%)	1 (3.8)	1 (4.2)	-	-
Features most consistent with an alternative diagnosis				
NSIP, n (%)	7 (26.9)	14 (58.3)	10 (52.6)	3 (21.4)
Non-NSIP, n (%)	1 (3.8)	2 (8.3)	-	1 (7.1)

AD: autoimmune diseases; DLCO: diffusing capacity of the lungs for carbon monoxide; FEV1: forced expiratory volume in one second; FVC: forced vital capacity; HRCT: high-resolution computed tomography; ILD: interstitial lung disease; NSIP: non-specific interstitial pneumonia; SD: standard deviation; UIP: usual interstitial pneumonia.
